# Adherence to guidelines for antimictrobial prophylaxis in surgery in university hospitals in Serbia

**DOI:** 10.1186/1753-6561-5-S6-P152

**Published:** 2011-06-29

**Authors:** G Ćosić, B Jovanović, O Horvat, A Sabo

**Affiliations:** 1 Department for Infection Control and Prevention, Institute of Public Health of Vojvodina, Novi Sad, Serbia; 2 Hospital Epidemiology, Clinical Center of Serbia, Belgrade, Serbia; 3 Department of Pharmacology, Toxicology and Clinical Pharmacology, Medical Faculty Novi Sad, Novi Sad, Serbia

## Introduction / objectives

The aim of the paper was to evaluate the adherence of surgeons to guidelines for antimicrobial prophylaxis in two largest university hospitals in Serbia.

## Methods

A study was performed in October 2010 in a General Surgery Clinic in a hospital in Belgrade and in February 2011 in seven surgery clinics in a hospital in Novi Sad. Data concerning preoperative health status, type and duration of surgery and parameters of antimicrobial prophylaxis (drug choice, timing of first dose, number of dosis and duration of prophylaxis) were collected. Rresults are given as frequency and percent. Consumption of antibiotics was expressed as DDD/100 patient days, that was calculated using ATC/DDD methodology.

## Results

A total of 312 patients were enrolled and operated on electively. In a hospital in Belgrade the most frequently prescribed antibiotic was ceftriaxone (33%), more than half of patients received antibiotics longer than suggested in the protocol and there was no data on timing of first dose available. In a hospital in Novi Sad the total consumption of antibiotics for prophylactic use was 25.6 DDDs/100 patient days. Cephalosporins with 59.5% were most commonly utilized (out of them cefuroxime with 12.3 DDDs/100 patient days was most commonly used), followed by aminoglycosides with 14.5% (out of them gentamicin with 3.8 DDDs/100 patient days), while ciprofloxacin was on the third place with 10.7%. Timing was appropriate in 44.3% of patients while the duration of prophylaxis was optimal in 28%.

## Conclusion

Interventions have to be made about adoption of adequate guidelines for surgical prophylaxis in both university hospitals in Serbia.

## Disclosure of interest

None declared.

